# Mid-Cretaceous amber inclusions reveal morphogenesis of extinct rachis-dominated feathers

**DOI:** 10.1038/s41598-019-54429-y

**Published:** 2019-12-02

**Authors:** Nathan R. Carroll, Luis M. Chiappe, David J. Bottjer

**Affiliations:** 1 The Dinosaur Institute, Natural History Museum of Los Angeles County, Los Angeles, CA 90007 USA; 2 Department of Earth Sciences, University of Southern California, Los Angeles, California 90089-0740 USA

**Keywords:** Epithelial-mesenchymal transition, Evolutionary developmental biology, Palaeontology

## Abstract

We describe three-dimensionally preserved feathers in mid-Cretaceous Burmese amber that share macro-morphological similarities (e.g., proportionally wide rachis with a “medial stripe”) with lithic, two-dimensionally preserved rachis-dominated feathers, first recognized in the Jehol Biota. These feathers in amber reveal a unique ventrally concave and dorsoventrally thin rachis, and a dorsal groove (sometimes pigmented) that we identify as the “medial stripe” visible in many rachis-dominated rectrices of Mesozoic birds. The distally pennaceous portion of these feathers shows differentiated proximal and distal barbules, the latter with hooklets forming interlocking barbs. Micro-CT scans and transverse sections demonstrate the absence of histodifferentiated cortex and medullary pith of the rachis and barb rami. The highly differentiated barbules combined with the lack of obvious histodifferentiation of the barb rami or rachis suggests that these feathers could have been formed without the full suite and developmental interplay of intermediate filament alpha keratins and corneous beta-proteins that is employed in the cornification process of modern feathers. This study thus highlights how the development of these feathers might have differed from that of their modern counterparts, namely in the morphogenesis of the ventral components of the rachis and barb rami. We suggest that the concave ventral surface of the rachis of these Cretaceous feathers is not homologous with the ventral groove of modern rachises. Our study of these Burmese feathers also confirms previous claims, based on two-dimensional fossils, that they correspond to an extinct morphotype and it cautions about the common practice of extrapolating developmental aspects (and mechanical attributes) of modern feathers to those of stem birds (and their dinosaurian outgroups) because the latter need not to have developed through identical pathways.

## Introduction

**Figure 1:**
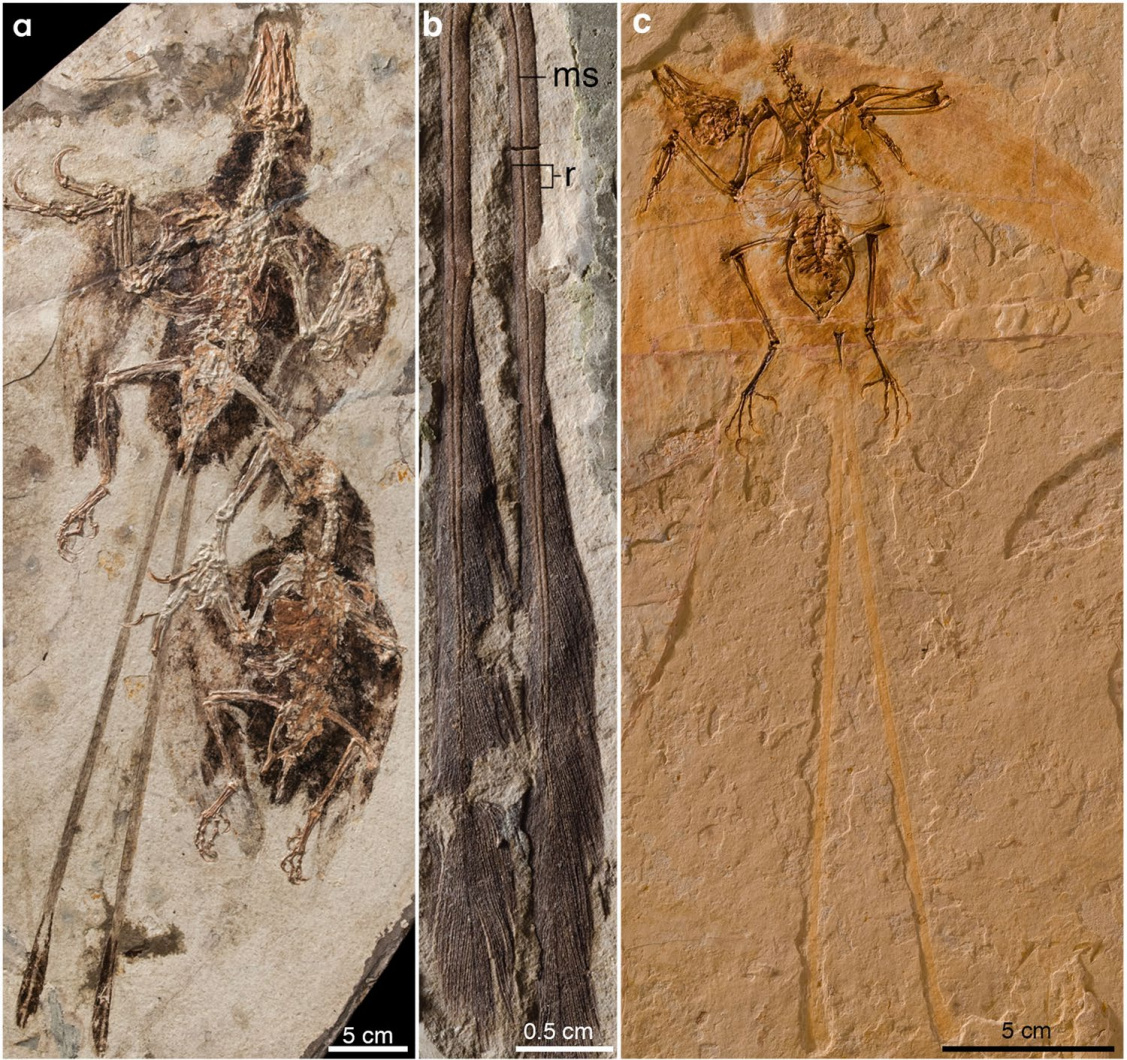
Lithic Preservation of Early Cretaceous rachis-dominated feathers from the Yixian Formation. (**a**) *Confuciusornis* pair with and without rectrices. (**b**) Paired rectrices of an indeterminate enantiornithine displaying limited 3D preservation. (**c**) *Junornis;* an enantiornithine with proportionately long rachis-dominated rectrices. Abbreviations: ms, medial stripe; r, rachis.

Exceptional fossil feather preservation in lithic *Lagerstätten*, largely from the Lower Cretaceous, has led to the recognition of several purportedly unique feather types that are unknown in modern birds^1–6^. Although some of these unique morphotypes may have been altered taphonomicaly^7,8^, the rachis dominated feather (RDF) is a commonly reported and morphologically consistent type of extinct feather, likely representing a true morphology^9^. A clear definition for the morphology of RDFs has yet to be fully proposed, largely due to disagreement in how to interpret the features exhibited. In general, these feathers have an elongate “racket-plume” appearance consisting of a long proximal portion formed by a proportionally wide rachis, a distally vaned section, and a dark “medial stripe” that runs the full length of the feather (Fig. 1). Although many of these feathers are exquisitely preserved with some structurally resistant components (i.e., melanosomes) observable at the microscale^10^, the preservation of these two-dimensional lithic fossils prevents an accurate three-dimensional microstructural whole-feather assessment. Without three-dimensional, contiguous micro to macro scale preservation, it is difficult to make accurate assessments of the detailed morphology of these feathers, let alone their development, therefore hindering comparisons with modern feather development. This is typified by differing interpretations of the “medial stripe” as representing the rachis of a vaned feather with undifferentiated barbs^11^ or a trait (e.g., longitudinal groove) within a broader rachis^6,12–14^.

As the number of reported RDFs has increased, so have the reported variations in morphology and preservation. The greatest disparity is related to the extent of the vaned portion, with pennaceous vanes running the full length of the feather in the Chinese Early Cretaceous enantiornithines *Parapengornis*^15^ and *Eopengornis*^16^, and more distally restricted in most other coeval taxa^17,18^. Likewise, the shape of the distally restricted pennaceous portion varies between taxa (and sometimes intraspecifically), ranging from variably racket-shaped in the basal pygostylian *Confuciusornis*, and the enantiornithines *Paraprotopteryx* and *Dapingfangornis*, to morphologies in which the differentiation of the vanes is more gradual (e.g., Brazilian enantiornithine *Cratoavis*)^16,19^. Furthermore, the “medial stripe”, observable on a number of Early Cretaceous specimens as a thin, pigmented line or a longitudinal groove^19^, is not apparent in some specimens^10^. These morphologies, all documented in lithic fossils, are clearly subject to taphonomic alteration, and the degree by which these biases modify morphology is not fully known^20^. Regardless of these shortcomings, the nature of these unique feathers has been the center of much debate.

In recent years, spectacular discoveries of avian fossils and putative non-avian dinosaurs in mid-Cretaceous amber from northern Myanmar have provided important three-dimensional and micro-structural information about Mesozoic feathers^21–23^. Among these new findings are a number of feathers with morphologies that closely match that of RDFs at the macroscale, with preservation of features down to the microscale. A combination of transmitted light microphotography and micro-CT scanning was used to elucidate important three-dimensional, microscale features previously unattainable from the lithic record. Here we present a set of new amber specimens to identify previously unknown features of RDFs and highlight differences in the development of these feathers when compared to their modern counterparts.

## Results

**Figure 2:**
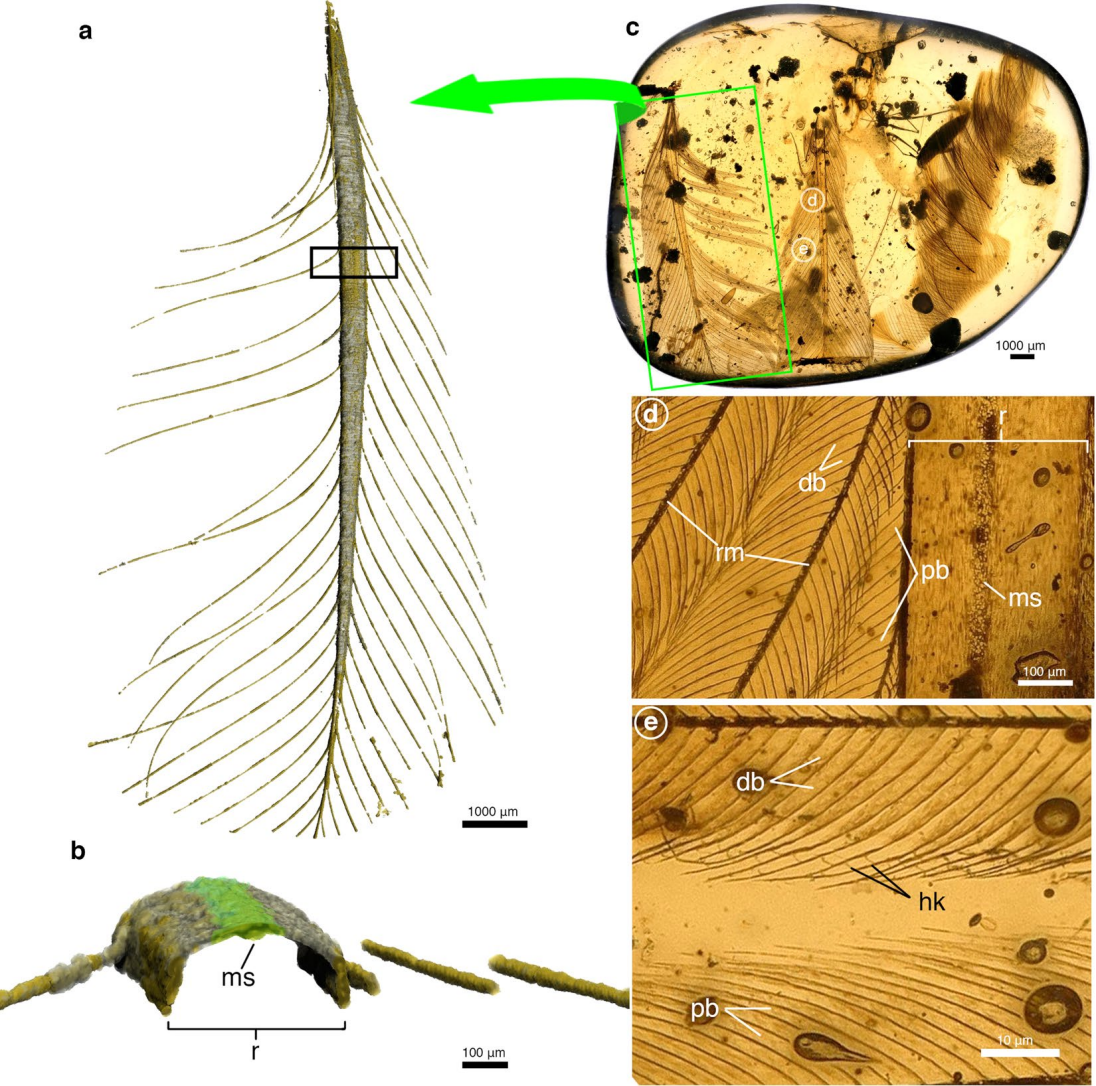
Paired rachis-dominated feathers preserved in amber. (**a**) X-ray μCT rendering of rachis-dominated feather in dorsal view preserved in LACMDI 158032. (**b**) Digitally sectioned segment of rachis from boxed region in (**a**) with medial stripe highlighted in green. (**c**) Whole specimen view of LACMDI 158032 with paired rectrices in ventral view. (**d**) Ventral view of rachis and barbs from location indicated in (**c**). (**e**) Ventral view of separated barbs from location indicated in (**c**). Abbreviations: db, distal barbule; hk, hooklets; ms, medial stripe; r, rachis; rm, ramus.

**Figure 3:**
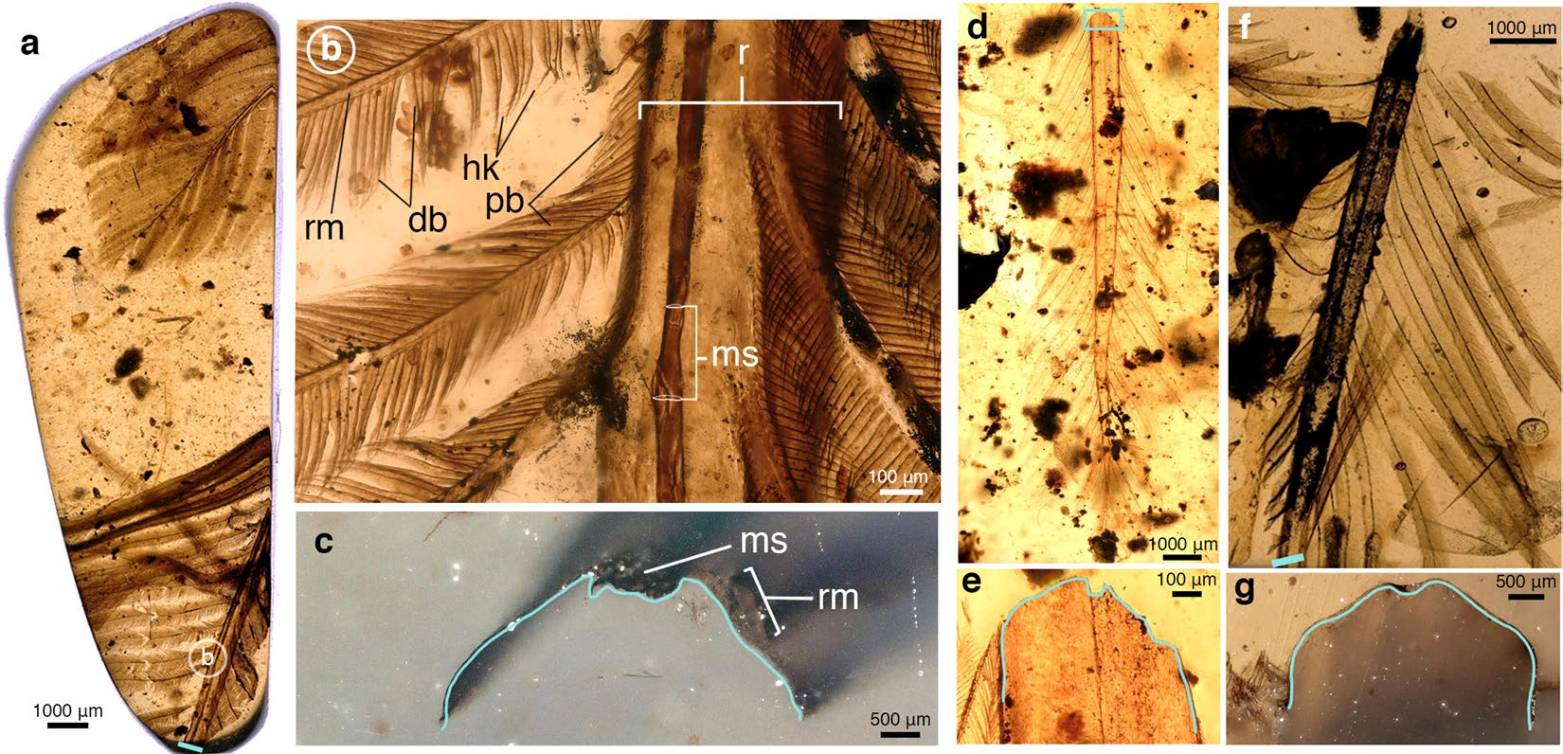
Rachis-dominated feathers in amber. (**a**) LACMDI 158557 with two partial feathers in dorsal view (**b**) Magnified dorsal view of feather from region in (**a**) showing rachis, medial stripe, barbs, and barbules. (**c**) Transverse view from blue bar in (**a**) of truncated distal rachis. Blue line traces the ventral surface profile of the rachis. (**d**) LACMDI 158490 in ventral view. (**e**) Transverse view from box in (d) of truncated proximal rachis with blue line tracing the ventral surface profile. (**f**) LACMDI 158555 in ventral view. (**g**) Transverse view from box in F of truncated distal rachis with blue line tracing the ventral surface profile. Abbreviations: db, distal barbule; hk, hooklets; ms, medial stripe; r, rachis; rm, ramus.

Our study is based on feathers preserved in 5 amber specimens (Figs. 2 and 3) in the collection of the Natural History Museum of Los Angeles County’s Dinosaur Institute (LACMDI). These specimens are from the Tanai Township (Myitkyina District) in Myanmar’s Kachin Province, and they date to the middle Cretaceous (98.8 ± 0.6 Ma)^21^. The pennaceous feathers contained in these amber pieces range in total preserved length from 23 mm to 6.5 mm, and display minor variations in the details of their morphology and preservation. While some of these feathers are isolated (LACM-DI 158490, 158555), most occur alongside other feather synclusions. In the case of LACM-DI 158032 (Fig. 2c) the feathers of interest are preserved as parallel-aligned pairs.

Despite the variation in size and preservation, these feathers share a common rachis construction. In planar view, the rachis is proportionally wide and bears a “darkened” medial stripe running its full preserved length; this characteristic is observable with transmitted light in all studied specimens (Figs. 2c,d and 3a,d,f). It should be noted that due to the back-lit nature of amber photomicrographs, it is difficult to discern whether darkening is due to a thickened keratin cortex, pigmentation, or a combination of both factors. The dorsal surface of the rachis is slightly convex with a dorsal groove that corresponds to the medial stripe. Ventrally, the concave surface mirrors the convex dorsal surface, with a longitudinal medial ridge consequent with the medial groove of the dorsal surface. Transverse cross sections of the rachis reveal an overall ventrally concave morphology defined by an incredibly thin cortex (Fig. 2b). The cortex is thinnest in the areas lateral to the medial stripe but medial to where the barbs attach, with thicknesses between specimens ranging from approximately 3 μm (LACMDI 158555) to 10 μm (LACMDI 158205). The cortical thickness does not appear to vary along the preserved proximal-distal axis. The lateral walls, where the barbs attach to the rachis, achieve the greatest cortical thickness, ranging approximately between 10–36 μm (Figs. 2a and 3b,c,e). The dorsal groove, present in all specimens, is variable in the details of its morphology and preservation. In the case of LACMDI 158032, the groove is shallow, with a granulated texture distinct from the longitudinally aligned fibrous texture of the rest of the rachis (Fig. 2d). In LACMDI 158557, a darkened strip of material lies within the dorsal groove and appears to be loosely adhered to the cortical surface. The dorsal groove maintains a constant width along the preserved length of the rachis, even as the overall width of the rachis tapers distally. In feathers where the distal-most tip is preserved (LACMDI 158205 and LACMDI 158557), the groove shallows and fades in prominence near the point where the rachis narrows to the width of the dorsal groove and is not observable at the distal-most tip of the feather.

The vanes of all feathers described here appear to be symmetric, although barb separation and truncation prevents accurate measurements of total vane width. The vanes are formed by interlocking barbs, which are comprised of a central ramus with distally and proximally projecting barbules (Fig. 2d,e). The thickness of the barb rami is comparably thin to that of the rachis (3–10 μm) and has a simple, ribbon-like morphology. The proximal and distal barbules share a similar morphology in their attaching base, which is a long narrow plate with a dorsal flange that connects with the ramus at an angle of 41–46° to the long axis of the barb. The bases of the distal barbules attach to the dorsal aspect of the distal face of the rami, while the bases of the proximal barbules attach to the ventral aspect of the proximal face of the rami. The proximal barbules continue their insertion proximally beyond the junction of the barb rami and the rachis as far as the insertion of the next proximal barb (Figs. 2d and 3b). The pennuli of the proximal barbules is demarcated by the presence of dorsal teeth and a tapering of the dorsoventral width. The pennuli of the distal barbules are marked by an abrupt increase in angle and the presence of weakly to strongly hooked barbicels that curve ventrally (Fig. 3b).

## Discussion

Because the feathers here described only include relatively short (and presumably distal) portions of vaned, RDFs, this study does not provide direct comparisons for the proximal, vane-less portions of the rachis described from lithic deposits. Nonetheless, the new fossils significantly expand our knowledge of the morphology of RDFs. In particular, these fossils show that the rachis of these feathers was (1) dorsoventrally extremely thin (albeit their incomplete preservation prevents determining if such condition extended throughout their entire length), (2) ventrally concave, and (3) scarred by a dorsal groove and a ventral ridge. Most notably, they also show that these feathers lack obvious medullary pith within the rachis or barb rami, an important structural and developmental component of a modern feather^24,25^. Our observations also indicate that the “medial stripe” visible in many RDFs preserved as lithic fossils corresponds to a dorsal, median groove that extended throughout the length of the rachis (Figs. 1b, 2b and 3). The fact that these traits are unknown among modern feathers indicates that as previously claimed^6^, the unique RDFs of many Mesozoic avians represent an extinct morphotype, which existence is thus far conclusively known from only the first half of the Cretaceous (interpretations of the Late Jurassic *Praeornis sharovi* as an RDF continue to be controversial^26^. Also of note are the differentiated proximal and distal barbules with definable bases and ciliated pennuli exhibited by these specimens.

The remarkably thin cross-sectional morphology of the rachises described here is a surprising departure from modern bird rachises, which are generally four-sided with a compact outer cortex encasing an inner medulla composed of spongey pith^24^. This “composite foam sandwich”^27^ construction is found even in specialized feathers that have a broadened rachis, such as the feathers of penguins^28^ or the specialized feather tips of various birds^29^. The functional significance of the extremely thin rachises described here is currently not understood, nor is it clear if this construction scales to the larger sizes more commonly reported from lithic fossils^6,12,16,26^.

**Figure 4:**
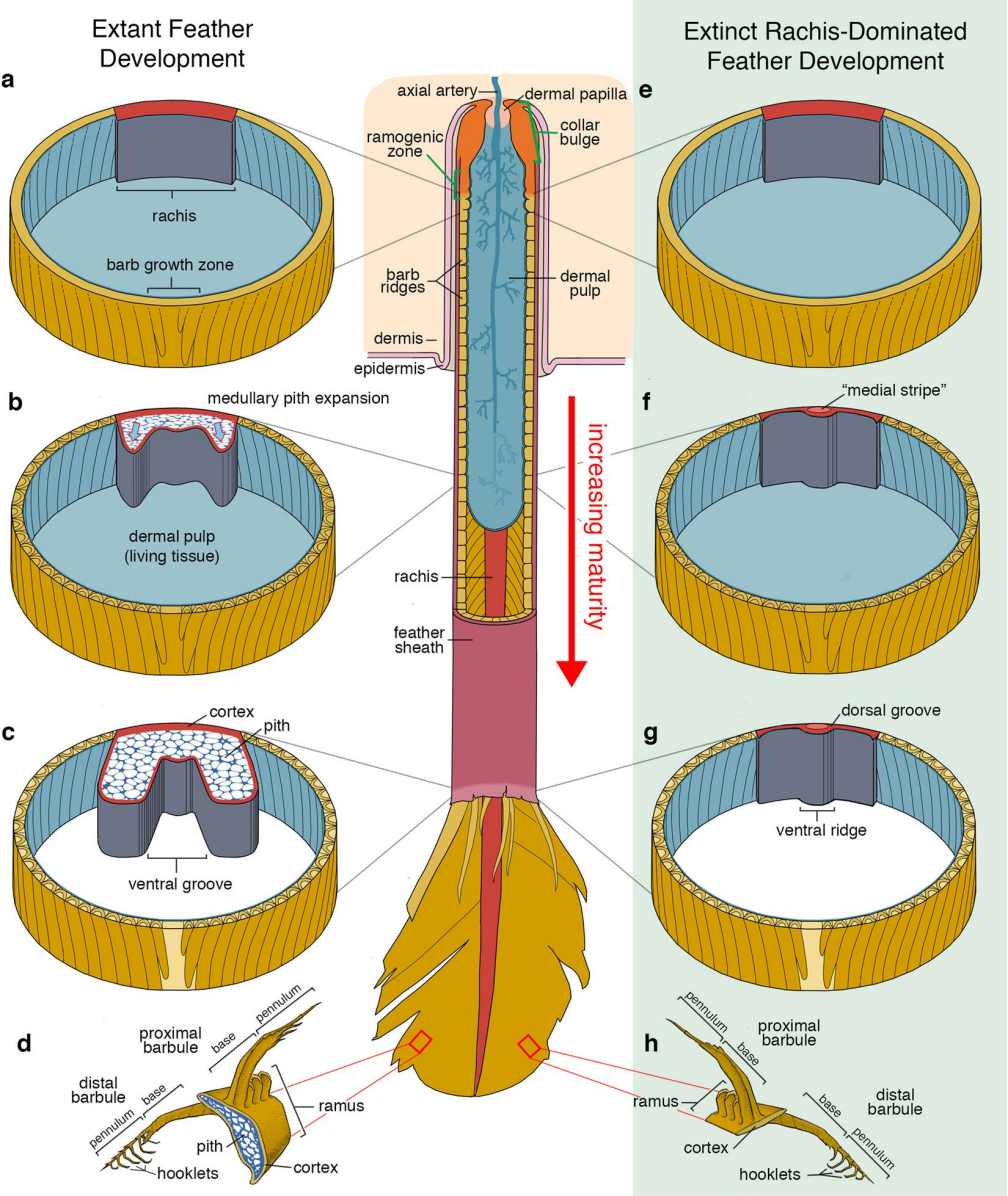
Generalized overview of the morphogenesis of a modern chicken tail feather and proposed morphogenesis of extinct rachis-dominated feather. (**a**–**c**) Cross sectional views of the developing epidermal feather tube of a modern chicken tail feather. Sections exhibit increasing maturity and cornification as the location moves distally. (**d**) Illustrated cut section of a mature barb from the deployed pennaceous vane of a modern chicken, modified from^24^. (**d**–**g**) Cross sectional views of the proposed developmental progression in the epidermal feather tube of an extinct rachis-dominated feather. (**h**) Illustrated cut section of a mature barb from the deployed pennaceous vane of rachis-dominated feather in amber.

To understand the significance of the morphologies observed in these amber preserved mid-Cretaceous feathers, it is useful to review the morphogenesis of a developing modern symmetrical pennaceous feather, with a focus on the components of the rachis and barb rami. Like all features of a feather, the rachis and barb rami develop from the tube of elongating epidermis by the proliferation of cells at the base of a feather follicle^30^. This initially undifferentiated epithelial cylinder begins branching in the ramogenic zone of the follicle through a complex spatio-temporal cell differentiation program in which some cells accumulate proteins that will form the body of the feather and others accumulate proteins that regulate the scaffolding function and programmed cell death of intermediate cells^31–33^. A cross section at the level of the ramogenic zone would reveal identifiable presumptive rachis and barb ridges (each ridge containing the ramus and barbules of a single barb) as the accumulation of various proteins begins (Fig. 4a). Subsequent morphogenetic events occur as the differentiating tube of cells moves distally and early-accumulating intermediate alpha-filament keratins (IF keratins) are rapidly replaced with beta-keratins and other specific proteins to complete the keratinocyte differentiation and eventual death^34^. The cells destined to form the rachis and barbs begin accumulating various “Corneous beta-proteins” (CbetaPs, sensu^34^). Mature feather cells, especially in barbs and barbules, are comprised almost entirely of CbetaPs, while detectibly more IF keratins are present in the calamus, ventral rachis, and ventral barb rami^32,35–38^. A cross section of the cornifying feather tube near the emergence of the epidermal surface would reveal the cells of the rachis and barb rami differentiating into the inner medulla and outer cortex (Fig. 4b). Medullary pith cell expansion within the rachis and rami extends the ventral aspects of these structures medially and laterally. This process ends as the cells fully cornify and the outer constraining sheath desiccates and is preened away, allowing for the feather to deploy (Fig. 4c). Of interest to this study is that the differentiation of cells destined to become the CbetaP rich dorsal and lateral aspects of the rachis, as well as those of the barbules, occurs earlier in the development of the feather than the ventral aspects of the rachis and barb rami^34^.

Although the exact molecular mechanisms by which the epithelial cells of the medulla vacuolate and expand are not well understood, recent work on the molecular basis of the distinctive mutant frizzle feather phenotype found in chickens provides important insight into this process. Ng *et al*.^36^ found that the rachis of frizzle feathers has a smaller medulla compared to the normal leghorn controls and suggest that the frizzle phenotype is caused by a defect in the ventral part of the rachis, via a misexpression of KRT75, an IF keratin. Their research found that KRT75 is expressed in the ventral part that is destined to become the medulla, whereas ß-keratin is expressed in the dorsal part of the rachis and ramus that is destined to become the cortex.

The unique morphology of the RDFs of many stem avian lineages is difficult to reconcile with our extensive knowledge about the development of modern feathers; the conservative developmental circuits that produce a modern feather do not regularly generate a feather with the morphology of these extinct feathers, namely one that lacks medullary pith and has the structural traits described for these feathers. The cross-sectional morphology of a modern rachis is reliant on the internal expansion of medullary pith as the feather cells mature and keratinize within the sheath^25^. The extremely thin nature of the RDFs described here, along with the absence of any medullary pith or structures known to rely on medullary pith development appears to necessitate a different developmental explanation (Fig. 4e–h).

The high degree of barbule differentiation combined with the lack of obvious histodifferentiation of the barb rami or rachis suggests that these feathers could have developed without the full suite and developmental interplay of IF keratins and CbetaPs that is employed in the cornification process of modern birds and could be produced with modifications to the subperiderm layer^31,34^. This hypothesis provides one alternative pathway (perhaps not the only one) of how the extinct RDFs could have developed. Clearly further studies are needed to test this hypothesis but the realization that certain extinct feathers are unlikely to have developed through identical developmental mechanisms that produce a modern feather cautions the use of modern developmental pathways and mechanical properties of feathers to infer aspects of the plumage of extinct, Mesozoic avians, a practice that has been common among researchers^39–41^. If the RDFs preserved as lithic fossils share the same construction and cross-sectional profile as RDFs in amber, as suggested from semi three-dimensional remains from China (Fig. 1b) and Brazil, then previous assumptions about their functional performance need to be reevaluated. The consistently straight, un-bent preservation of RDFs in lithic fossils^6,9,19^ is understandably suggestive of a rigid mechanical nature and is seemingly at odds with the extreme rachis construction described here. However, numerous feathers figured by Xing *et al*.^42^ from the same mid-Cretaceous deposit display consistent morphologies with those presented in this study, suggesting that this rachis construction was the dominate developmental pathway to produce RDFs in the mid-Cretaceous. Evaluating incongruences between inferred mechanical aspects of preserved RDFs from the entire Mesozoic in the light of the unexpected three-dimensional morphologies described here is outside of the scope of this study, but a greater understanding and consideration of the construction of RDFs in three dimensions at multiple scales will be critical in understanding their mechanical performance^41^. The excellent three-dimensional and micro-structural preservation in amber allows for unprecedented comparisons to modern feathers and can provide insight into extinct and extant feather development.

## Materials and Methods

Photomicrographs of all specimens were taken with a Keyence VHX-500 digital microscope with a transmitted light base. Focused images were created using the Keyence multiple image stacking software. One of the RDF inclusions of LACMDI 158032 was imaged using x-ray microtomography. The scan was conducted on a GE Phoenix Nanotom M microCT scanner at the Molecular Imaging Center, Department of Radiology, University of Southern California Keck School of Medicine using a current of 400 µA and voltage of 60 kV. A molybdenum target with no filter was used for the scan. The images were reconstructed from the raw dataset with voxel size of 3.5 µm. VGStudioMax 3.0 software was used for visualization, segmentation, and measurements. The feather was digitally isolated from the surrounding amber matrix by using a variety of threshold values with the “region growing” and other “region of interest” tools during the segmentation process, after applying a Gaussian filter. Original CT files of the segmented feather are available from the Dryad database at 10.5061/dryad.bzkh1894f upon request.
